# The Epigenetic Regulator EZH2 Instructs CD4 T Cell Response to Acute Viral Infection via Coupling of Cell Expansion and Metabolic Fitness

**DOI:** 10.1128/JVI.01627-20

**Published:** 2020-11-23

**Authors:** Ren Li, Zhiwei Pan, Jialin Wu, Shuai Yue, Yao Lin, Yang Yang, Zhirong Li, Li Hu, Jianfang Tang, Lingling Shan, Qin Tian, Peng Jiang, Ping Wei, Lilin Ye, Pinghuang Liu, Xiangyu Chen

**Affiliations:** a State Key Laboratory of Veterinary Biotechnology, Harbin Veterinary Research Institute, Chinese Academy of Agricultural Sciences, Harbin, Heilongjiang, China; b College of Veterinary Medicine, Northeast Agricultural University, Harbin, Heilongjiang, China; c Institute of Immunology, PLA, Third Military Medical University, Chongqing, China; d Department of Respiratory Disease, General Hospital of Xinjiang Military Command, Urumqi, Xinjiang, China; e Institute of Cancer, Xinqiao Hospital, Third Military Medical University, Chongqing, China; f Comparative Immunology Research Center, College of Veterinary Medicine, China Agricultural University, Beijing, China; Hudson Institute of Medical Research

**Keywords:** acute viral infection, EZH2, CD4 T cell expansion, mTOR, metabolism

## Abstract

The protection of a majority of viral vaccines is mediated by CD4 T cell-dependent humoral immunity. The methyltransferase enhancer of zeste homolog 2 (EZH2) dictates the differentiation of naive CD4 T cells into distinct effector T helper subsets at the onset of acute viral infection. However, whether and how EZH2 manipulates differentiated virus-specific CD4 T cell expansion remain to be elucidated. Here, we found that EZH2 is integral for virus-specific CD4 T cell expansion in a mouse model of acute viral infection. By a mechanism that involves fine-tuning the mechanistic target of rapamycin (mTOR) signaling, EZH2 participates in integrating metabolic pathways to support cell expansion. The genetic ablation of EZH2 leads to impaired cellular metabolism and, consequently, poor CD4 T cell response to acute viral infection. Thus, we identified EZH2 as a novel regulator in virus-specific CD4 T cell expansion during acute viral infection.

**IMPORTANCE** The CD4 T cell response is critical in curtailing viral infection or eliciting efficacious viral vaccination. Highly efficient expansion of virus-specific CD4 T cells culminates in a qualified CD4 T cell response. Here, we found that the epigenetic regulator EZH2 is a prerequisite for the virus-specific CD4 T cell response, with a mechanism coupling cell expansion and metabolism. Thus, our study provides valuable insights for strategies targeting EZH2 to improve the efficacy of CD4 T cell-based viral vaccines and to help treat diseases associated with aberrant CD4 T cell responses.

## Abstract (precis)

The CD4 T cell response is critical in curtailing viral infection or eliciting efficacious viral vaccination. Highly efficient expansion of virus-specific CD4 T cells culminates in a qualified CD4 T cell response. Here, we found that the epigenetic regulator EZH2 is a prerequisite for the virus-specific CD4 T cell response, with a mechanism coupling cell expansion and metabolism. Thus, our study provides valuable insights for strategies targeting EZH2 to improve the efficacy of CD4 T cell-based viral vaccines and to help treat diseases associated with aberrant CD4 T cell responses.

## INTRODUCTION

Histone modifications are essential in initiating epigenetic reprogrammings that prepare the activation, differentiation, proliferation, and effector function of CD4 T cells (1–3). One crucial regulator of histone modifications is enhancer of zeste homolog 2 (EZH2), which functions as a methyltransferase unit of the PRC2 complex that induces trimethylation of histone H3 at lysine 27 (H3K27me3). This modification silences the corresponding genes by recruiting chromatin-compressing proteins (4). EZH2 and associated H3K27me3 participate in eliciting differentiation and effector function of various CD4 T subsets, including T_H_1, T_H_2, regulatory CD4 T (T_reg_), and follicular helper CD4 T (T_FH_) cells (3, 5–9). In the T_H_1 and T_H_2 subsets, EZH2-H3K27me3 modification impedes T_H_1 and T_H_2 differentiation by directly inhibiting the transcription of lineage transcriptional factors (*Tbx21* and *Gata3*) and genes encoding lineage-specific cytokines (*Ifng* and *Il13*) (5, 6). In the T_reg_ lineage, EZH2 stabilizes the T_reg_-lineage transcriptional program by suppressing other T_H_ subset-specific genes (7). In the scenario of T_FH_ cells, we and other groups demonstrated that EZH2 is a prerequisite for early T_FH_ cell differentiation by stabilizing lineage-associated genes, especially *Bcl6* (8, 9).

In addition to epigenetic regulation, dramatic metabolic reprogramming also entails activated CD4 T cells characterized by high metabolic flux through growth-promoting pathways, thus satisfying the energy requirements of T cell differentiation, proliferation, and effector function (10). The mechanistic target of rapamycin (mTOR), a conserved serine/threonine kinase, is critical in coordinating growth-promoting pathways to support glycolysis, protein synthesis, fatty acid synthesis, and mitochondrial functions (11). The mTOR kinase forms two distinct complexes: mTOR complex 1 (mTORC1) and mTORC2, with shared mTORs but different scaffolding subunits (11). In the absence of mTOR signaling, naive CD4 T cells fail to differentiate into T_H_1, T_H_2, T_H_17, T_reg_, T_FH_, and follicular regulatory CD4 T cells (12–14).

Epigenetic modifications and metabolic alterations are highly intertwined (15). The CD4 T cell response to viral infection is the summation of antigen-induced epigenetic reprograming and metabolic shifts. However, the metabolic effects of epigenetic regulator EZH2 on virus-specific CD4 T cell responses have not been appreciated. By using an acute lymphocytic choriomeningitis virus (LCMV) infection model, we observed elevated expression of EZH2 in early-activated virus-specific CD4 T cells. The increase in EZH2 protein is mediated by T cell receptor (TCR) engagement and is required to initiate the expansion of virus-specific CD4 T cells. Mechanistically, EZH2 functions as a regulator of mTOR signal activity and thus coordinates pathways related to metabolic processes to fuel CD4 T cell expansion. Furthermore, the EZH2-mTOR axis supports the expansion of antigen-specific CD4 T cells during both primary and secondary CD4 T cell responses.

## RESULTS

### EZH2 is crucial for CD4 T cell response during acute viral infection.

**FIG 1:**
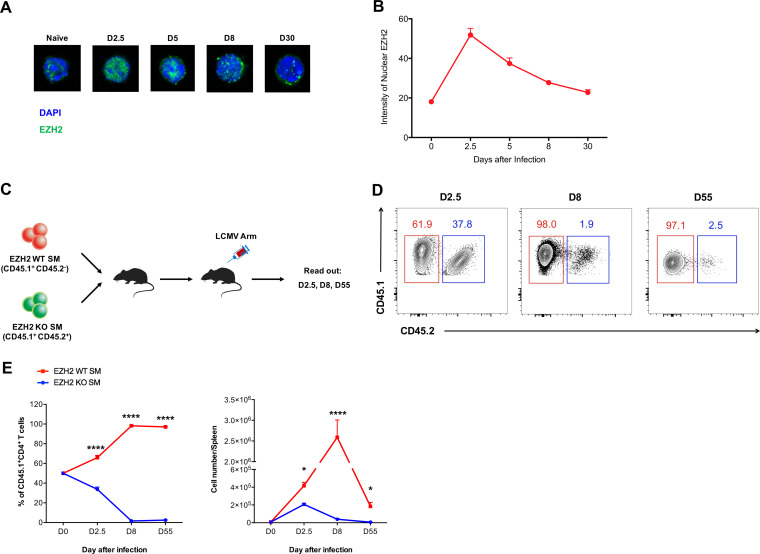
EZH2 is crucial for the CD4 T cell response during acute viral infection. (A and B) Confocal microscopy of EZH2 in SMARTA (SM) CD4 cells from the spleens of CD45.2^+^ wild-type mice receiving adoptive transfer of CD45.1^+^ SM cells at days 2.5, 5, 8, and 30 after LCMV Armstrong infection and in naive (CD44^lo^ CD62L^hi^) SM CD4 T cells. (C) Experimental setup. *Ezh2*^fl/fl^ SM cells (CD45.1^+^ CD45.2^−^; EZH2 WT SM) and *Ezh2*^fl/fl^*Cd4*-Cre SM cells (CD45.1^+^ CD45.2^+^; EZH2 KO SM) were cotransferred into WT recipients (CD45.1^−^ CD45.2^+^), which were assessed on days 2.5, 8, and 55 after LCMV Armstrong infection. (D) Flow cytometry of EZH2 WT and EZH2 KO SM CD4 T cells for experiments depicted in panel C. (E) Frequency (left) and total number (right) of transferred EZH2 WT and EZH2 KO SM CD4 T cells for experiments depicted in panel C. *, *P* < 0.05; ****, *P* < 0.0001 (one-way ANOVA, Tukey’s multiple-comparison test). Data are representative of two independent experiments with at least 9 cells per group (for B, error bars are standard deviations [SDs]) or at least 4 mice per group (for D and E; error bars are SDs.).

The CD4 T cell response is pivotal for curtailing viral infection. To investigate the role of EZH2 in the CD4 T cell response during acute infection, we first adoptively transferred naive LCMV-specific SMARTA (SM) cells, which express a transgenic T cell receptor specific for the LCMV glycoprotein epitope I-A^b^GP66-77, into wild-type (WT) C57BL/6J recipient mice and subsequently infected the recipients with LCMV Armstrong strain virus. At days 2.5, 5, 8, and 30 after infection, we sorted the transferred SM CD4 T cells from the spleens of chimeric recipients and analyzed their EZH2 expression levels by confocal microscopy. As indicated in Fig. 1A and B, the EZH2 protein reached peak levels at day 2.5 and then declined to a basal level at day 8, suggesting EZH2 might play a role in regulating virus-specific CD4 T cell responses during the early phase of an acute viral infection. To test this hypothesis, we bred *Ezh2*^fl/fl^ or *Ezh2*^fl/fl^*Cd4*-Cre mice with SM mice to probe the role of EZH2 in CD4 T cell responses to acute viral infection. Naive *Ezh2*^fl/fl^ SM (CD45.1^+^ CD45.2^−^) and *Ezh2*^fl/fl^*Cd4*-Cre SM (CD45.1^+^ CD45.2^+^) CD4 T cells were mixed at equal ratios and cotransferred into congenic WT recipients (CD45.1^−^ CD45.2^+^) that were subsequently infected with LCMV Armstrong (Fig. 1C). At day 2.5 postinfection, we observed that EZH2-deficient SM CD4 T cells were outcompeted by the EZH2-sufficient ones (Fig. 1C and D). To our surprise, the enrichment of EZH2-sufficient SM CD4 T cells was more pronounced at day 8 after infection, and this pattern was maintained until day 55 postinfection (memory phase). Thus, our results pinpointed EZH2 as a crucial hub in fostering the CD4 T cell response in the early expansion phase during acute viral infection.

### Efficient CD4 T cell expansion is coordinated by EZH2.

**FIG 2:**
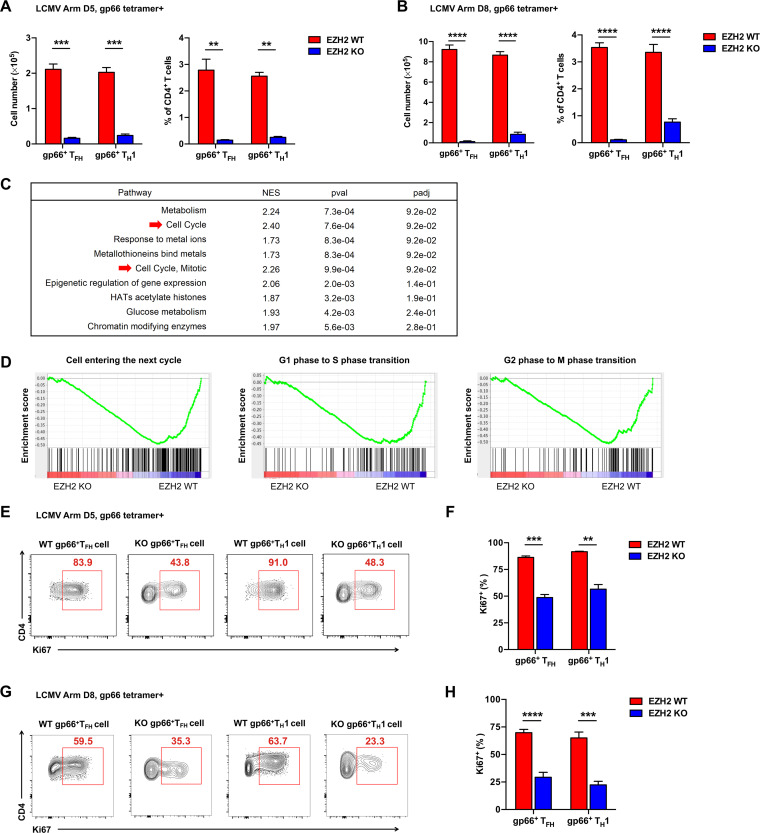
Efficient CD4 T cell expansion is coordinated by EZH2. Total number (left) and frequency (right) of GP66 tetramer^+^ T_FH_ and T_H_1 cells from the spleens of *Ezh2*^fl/fl^ (EZH2 WT) or *Ezh2*^fl/fl^*Cd4*-Cre (EZH2 KO) mice on day 5 (A) or day 8 (B) after infection. (C) Unbiased GSEA analysis of gene signatures between EZH2 WT and EZH2 KO CD4 T cells at day 8 after LCMV Armstrong infection (GEO accession code GSE110458). (D) GSEA analysis of gene signatures for cell cycling between EZH2 WT and EZH2 KO CD4 T cells at day 8 after LCMV Armstrong infection (GEO accession code GSE110458). (E) Flow cytometry analysis of GP66 tetramer^+^ T_FH_ and T_H_1 cells from the spleens of EZH2 WT or EZH2 KO mice described for panel A. Numbers adjacent to the outlined areas indicate the proportions of Ki67-positive cells, which are summarized in F. (G) Flow cytometry analysis of GP66 tetramer^+^ T_FH_ and T_H_1 cells from the spleens of EZH2 WT or EZH2 KO mice described for panel B. Numbers adjacent to the outlined areas indicate the proportions of Ki67-positive cells, which are summarized in H. **, *P* < 0.01; ***, *P* < 0.001; ****, *P* < 0.0001 (unpaired two-tailed *t* test). Data are representative of two independent experiments with at least 4 mice per group (for A, B, F, and H, error bars are standard errors of the means [SEMs]).

To further confirm the role of EZH2 in an endogenous system, *Ezh2*^fl/fl^ and *Ezh2*^fl/fl^*Cd4*-Cre mice were infected with LCMV Armstrong and analyzed at day 5 or day 8 postinfection. In response to acute viral infection, virus-specific CD4 T cells differentiate into either CXCR5-positive follicular helper T (T_FH_) cells or CXCR5-negative T_H_1 cells (16–18). As expected, the number and frequency of both virus-specific T_FH_ and T_H_1 cells were dramatically lower the in *Ezh2*^fl/fl^*Cd4*-Cre group (EZH2 KO) than in *Ezh2*^fl/fl^ group (EZH2 WT) at the early phase (Fig. 2A) and late phase (Fig. 2B) of the CD4 T cell response. To identify the potential mechanism(s) by which EZH2 disruption abolished the CD4 T cell response, we performed an unbiased functional gene set enrichment analysis (GSEA) with published data (8) and found that EZH2-sufficient CD4 T cells have an advantage over EZH2-deficient ones in the enrichment of gene signatures of “cell cycle” (normalized enrichment score [NES] = 2.40) and “cell cycle, mitotic” (NES = 2.26) (Fig. 2C). Furthermore, gene signatures of various phases of cell cycle, including “cell entering the next cycle” (NES = −1.28), “G_1_ phase to S phase transition” (NES = −1.19), and “G_2_ phase to M phase transition” (NES = −1.30) were noticeably enriched in EZH2-sufficient CD4 T cells (Fig. 2D). In line with the GSEA results, virus-specific CD4 T_FH_ and T_H_1 cells showed decreased proliferation in the absence of EZH2, as reflected by the cardinal proliferation marker Ki67 (Fig. 2E to H). Collectively, these data demonstrated that an abundance of EZH2 protein accelerates early-activated CD4 T cell expansion in acute viral infection.

### EZH2 deficiency blunts mTOR signaling in CD4 T cells.

**FIG 3:**
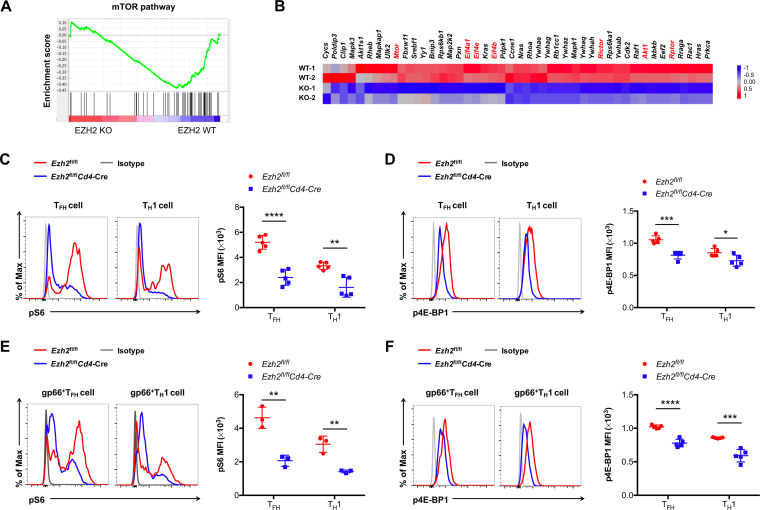
EZH2 deficiency blunts mTOR signaling in CD4 T cells during acute viral infection. (A) GSEA analysis of gene signatures for the mTOR pathway between EZH2 WT and EZH2 KO CD4 T cells on day 8 after LCMV Armstrong infection (GEO accession code GSE110458). (B) Heat map showing the mTOR signaling-related genes between EZH2 WT and EZH2 KO CD4 T cells. Flow cytometry analysis of phosphorylated (p)S6 (C) and p4E-BP1 (D) expression levels in T_FH_ and T_H_1 cells from spleens of EZH2 WT and EZH2 KO groups on day 8 after LCMV infection. Flow cytometry analysis of pS6 (E) and p4E-BP1 (F) expression levels in GP66 tetramer^+^ T_FH_ and T_H_1 cells from spleens of EZH2 WT and EZH2 KO groups on day 8 after LCMV infection. *, *P* < 0.05; **, *P* < 0.01; ***, *P* < 0.001; ****, *P* < 0.0001 (unpaired two-tailed *t* test). Data are representative of two independent experiments with at least 5 mice per group (for C, D, E, and F, error bars are SEMs).

It is well established that the mammalian target of rapamycin (mTOR) integrates environmental cues as an important means of regulating cell expansion (11). To explore whether the regulation of CD4 T cell expansion by EZH2 is coupled to mTOR signaling, we conducted further GSEAs for gene signatures related to the mTOR pathway with the aforementioned published data (8). Indeed, a noticeable bias was detected in EZH2 WT but not EZH2 KO CD4 T cells with regard to the mTOR pathway (Fig. 3A) (NES = −1.70, normalized *P* < 0.01) (Fig. 3A). Specifically, genes known to be important in mTOR signaling, including *Mtor*, *Rptor*, *Rictor*, *Akt1*, *Eif4a1*, etc., were selectively enriched in EZH2 WT CD4 T cells compared to that in EZH2 KO ones (Fig. 3B). Next, we set out to characterize the mTOR signaling in EZH2-sufficient and -deficient CD4 T cells. At day 8 after infection, we observed a severe reduction in the phosphorylation of S6 and 4E-BP1 (key targets downstream of mTOR complex 1 [mTORC1]) in T_FH_ and T_H_1 cells under the condition of EZH2 loss (Fig. 3C to F). Together, these results indicated that impaired mTOR activity contributes to the poor expansion of virus-specific CD4 T cells devoid of EZH2 protein upon viral infection.

### EZH2 is indispensable for the metabolic fitness of CD4 T cells in acute viral infection.

**FIG 4:**
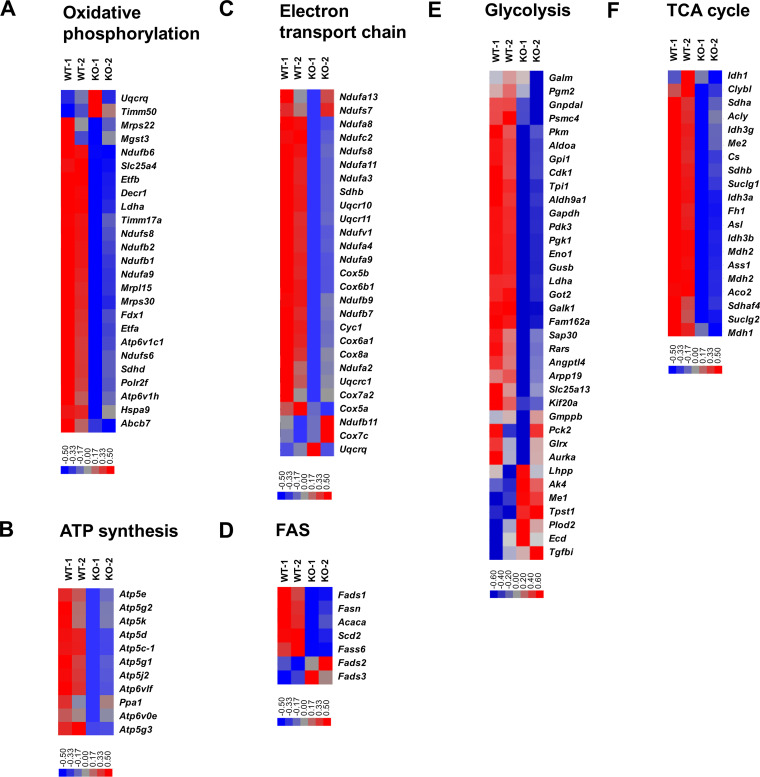
EZH2 is required for CD4 T cells to maintain cellular metabolism and energy homeostasis. (A-F) heat maps of genes selected in metabolic processes, including oxidative phosphorylation (A), ATP synthases (B), electron transport chain (C), FAS (D), glycolysis (E) and TCA cycle (F) between EZH2-sufficient and -deficient CD4 T cells. Data are analyzed based on published resource (GSE110458).

**FIG 5:**
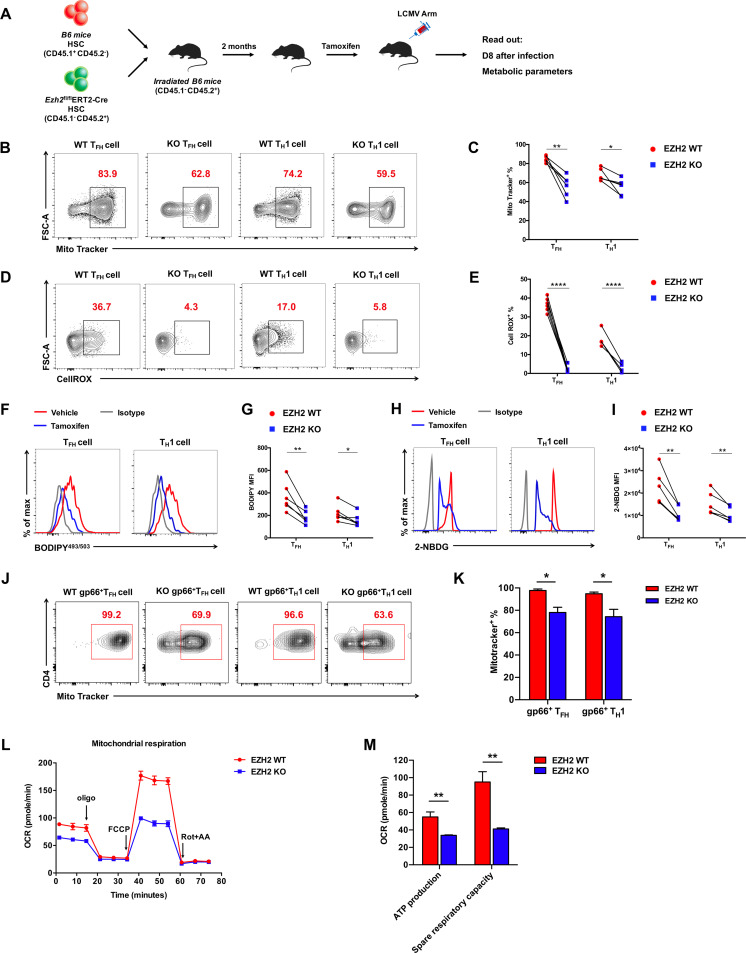
EZH2 is indispensable for CD4 T cell metabolic fitness. (A) Setup of bone marrow (BM) chimera experiment. Irradiated CD45.1^+^ WT B6 recipients were given adoptive transfer of CD45.1^+^ B6 BM cells (EZH2 WT) and CD45.2^+^ *Ezh2*^fl/fl^ERT2-cre BM cells (EZH2 KO) at ratio of 6:4. After 2 months of reconstitution, the recipients were treated with tamoxifen, infected with LCMV Armstrong, and analyzed on day 8 postinfection. (B) Flow cytometry analysis of MitoTracker-positive T_FH_ and T_H_1 cells for experiments described in panel A. Numbers adjacent to the outlined areas indicate MitoTracker-positive proportion of cells, which are summarized in C. (D) Flow cytometry analysis of CellROX-positive T_FH_ and T_H_1 cells in experiments described in panel A. Numbers adjacent to the outlined areas indicate CellROX-positive proportion of cells, which are summarized in E. Flow cytometry analyses of BODIPY^493/503^ (F and G) and 2-NBDG (H and I) in T_FH_ and T_H_1 cells in experiments described in panel A. (J) Flow cytometry analysis of MitoTracker-positive GP66 tetramer^+^ T_FH_ and T_H_1 cells from the spleens of *Ezh2*^fl/fl^ (EZH2 WT) or *Ezh2*^fl/fl^*Cd4*-Cre (EZH2 KO) mice on day 8 after LCMV Armstrong infection. Numbers adjacent to the outlined areas indicate MitoTracker-positive proportions of cells, which are summarized in K. (L) The oxygen consumption rate (OCR) analysis of CD4 T cells from the spleens of *Ezh2*^fl/fl^ (EZH2 WT) or *Ezh2*^fl/fl^*Cd4*-Cre (EZH2 KO) mice on day 8 after LCMV Armstrong infection. (M) Summary of ATP production and spare respiratory capacity between EZH2 WT and EZH2 KO CD4 T cells. *, *P* < 0.05; **, *P* < 0.01; ****, *P* < 0.0001 (paired two-tailed *t* test). Data are representative of two independent experiments with at least 6 mice per group (for C, E, G, I, K, L, and M, error bars are SEMs).

When activated, mTORC1 orchestrates mitochondrial oxidative metabolism, glucose metabolism, and lipid synthesis to support the energetic demands and cellular components needed for extensive CD4 T cell expansion (10). Recalling the fundamental role of EZH2 in mTORC1 activity, we next determined the impact of EZH2 in CD4 T cell metabolism. Indeed, an array of genes involved in oxidative phosphorylation (Fig. 4A), ATP synthases (Fig. 4B), the electron transport chain (Fig. 4C), fatty acid synthesis (FAS) (Fig. 4D), glycolysis (Fig. 4E), and the tricarboxylic acid (TCA) cycle (Fig. 4F) were specifically downregulated in EZH2-deficient CD4 T cells. Then, we set out to delineate the effects of EZH2 on the functional metabolic programs. To compare the metabolic activities of EZH2-sufficient and -deficient CD4 T cells under the same microenvironment, we generated bone marrow (BM) chimeric mice by mixing BM cells from *Ezh2*^fl/fl^ERT2-Cre mice (CD45.1^−^ CD45.2^+^) and BM cells from WT C57BL/6J mice (CD45.1^+^ CD45.2^−^) and injected the BM mixture into irradiated WT C57BL/6J recipients (CD45.1^+^ CD45.2^−^). Two months later, the BM chimeras were treated with tamoxifen, infected with LCMV Armstrong, and analyzed at day 8 postinfection (Fig. 5A). First, we found a reduction of mitochondrial contents in CD4 T_FH_ and T_H_1 cells under the condition of EZH2 deficiency (Fig. 5B and C), as indicated by MitoTracker staining (19). In addition, mitochondrial respiration (indicated by reactive oxygen species [ROS] expression [20]) was severely affected in EZH2-deficient CD4 T cells compared to that in EZH2-sufficient CD4 T cells (Fig. 5D and E). Meanwhile, we also observed largely compromised lipid synthesis (BODIPY^493/503^ staining [21]) (Fig. 5F and G) and glucose uptake (2-NBDG staining [22]) (Fig. 5H and I) in CD4 T cells in the absence of EZH2. Specifically, reduced mitochondrial content (Fig. 5J and K) and consequently dampened mitochondrial respiration (including ATP production and spare respiratory capacity) (Fig. 5L and M) were also observed in virus-specific CD4 T cells originating from *Ezh2*^fl/fl^*Cd4*-Cre mice compared to those from control mice. Taken together, the data indicated that EZH2 acts as an indispensable regulator to preserve metabolic fitness by coordinating mitochondrial respiration, glycolysis, and fatty acid synthesis in CD4 T cells during acute viral infection.

### The EZH2-mTOR axis is activated by TCR engagement.

**FIG 6:**
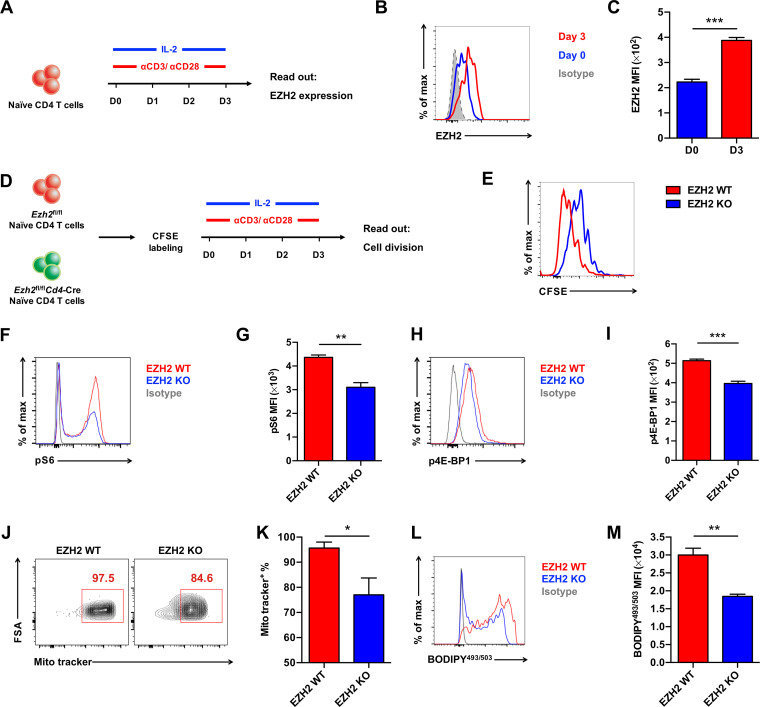
The EZH2-mTOR axis is activated by TCR engagement. (A) Experimental setup. Naive CD4 T cells from the spleens of C57BL/6J mice were cultured *in vitro* with αCD3/αCD28 antibodies and IL-2. (B) Flow cytometry analysis of EZH2 protein expression levels in CD4 T cells at day 0 and day 3 in culture, which are summarized in C. (D) Experimental setup. Naive CD4 T cells from the spleens of *Ezh2*^fl/fl^ (EZH2 WT) or *Ezh2*^fl/fl^*Cd4*-Cre (EZH2 KO) mice were first labeled with CFSE. Next, CFSE-labeled CD4 T cells were cultured *in vitro* and analyzed on day 3. (E) Flow cytometry analysis of CFSE-labeled CD4 T cells described in D. (F) Flow cytometry analysis of pS6 expression in CD4 T cells in experiments described in panel D, which are summarized in G. (H) Flow cytometry analysis of p4E-BP1 expression of CD4 T cells in experiments described in panel D, which are summarized in I. (J) Flow cytometry analysis of MitoTracker-positive CD4 T cells in experiments described in panel D. Numbers adjacent to the outlined areas indicate MitoTracker-positive proportion of cells, which are summarized in K. (L) Flow cytometry analysis of BODYPY^493/503^ expression levels in CD4 T cells in experiments described in panel D, which are summarized in M. *, *P* < 0.05; **, *P* < 0.01; ***, *P* < 0.001 (paired two-tailed *t* test). Data are representative of two independent experiments (for C, G, I, K, and M, error bars are SEMs).

T cell receptor (TCR) engagement leads to the activation and proliferation of CD4 T cells. To decipher the role of TCR engagement in regulating the EZH2-mTOR axis, we first assessed EZH2 protein expression upon αCD3/αCD28 stimulation (Fig. 6A). Consistent with previous reports (8, 23), upregulation of EZH2 protein expression was boosted by TCR engagement (Fig. 6B and C). Under the condition of EZH2 ablation, the proliferation of CD4 T cells was significantly delayed *in vitro*, as evidenced by a carboxyfluorescein succinimidyl ester (CFSE) dye dilution assay (Fig. 6D and E). Furthermore, mTOR signaling (Fig. 6F to I) and metabolic activities (Fig. 6J to M) of CD4 T cells were largely disrupted in the absence of EZH2 protein. These *in vitro* data, together with the aforementioned *in vivo* results, highlighted the key function of TCR engagement in initiating the EZH2-mTOR axis in CD4 T cells.

### The EZH2-mTOR axis is not required for the maintenance of late-differentiated or memory CD4 T cells.

**FIG 7:**
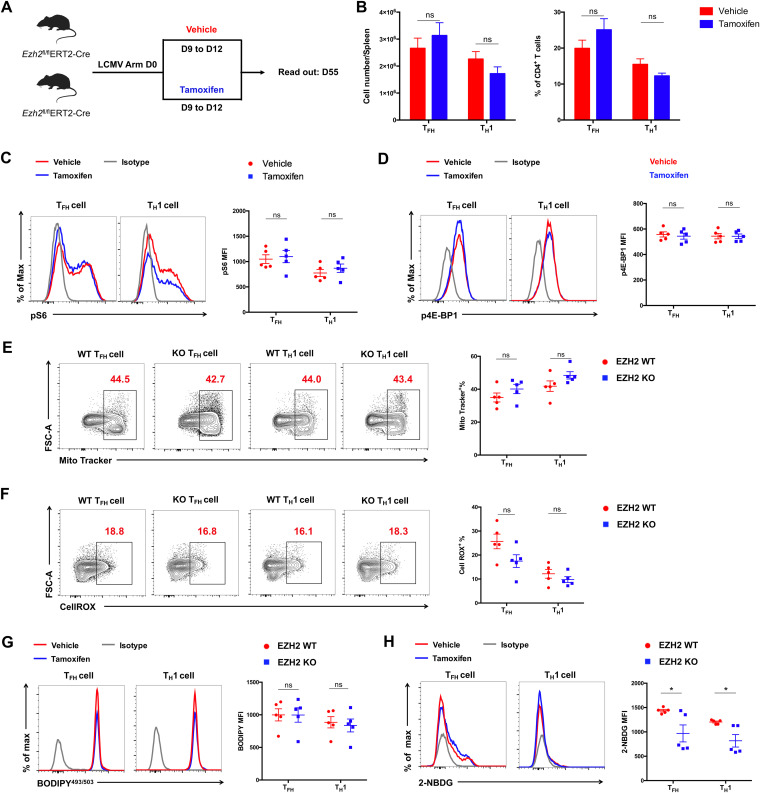
The EZH2-mTOR axis is not required for the maintenance of late-differentiated or memory CD4 T cells. (A) Experimental setup. *Ezh2*^fl/fl^ERT2-Cre mice were infected with LCMV Armstrong followed by administration of vehicle (EZH2 WT) or tamoxifen (EZH2 KO) from day 9 to day 12 and analyzed at day 55. (B) Total number (left) and frequency (right) of T_FH_ and T_H_1 cells from the spleens of vehicle-treated or tamoxifen-treated mice in experiments described in panel A. (C) Flow cytometry analysis of pS6 expression levels in T_FH_ and T_H_1 cells from the spleens of vehicle-treated or tamoxifen-treated mice in experiments described in panel A. (D) Flow cytometry analysis of p4E-BP1 expression levels in T_FH_ and T_H_1 cells from spleens of EZH2 WT and EZH2 KO groups in experiments described in panel A. (E) Flow cytometry analysis of MitoTracker-positive T_FH_ and T_H_1 cells from the spleens of vehicle-treated or tamoxifen-treated mice in experiments described in panel A. Numbers adjacent to the outlined areas indicate MitoTracker-positive proportion of cells and are summarized in the graph on the right. (F) Flow cytometry analysis of CellROX-positive T_FH_ and T_H_1 cells from the spleens of vehicle-treated or tamoxifen-treated mice in experiments described in panel A. Numbers adjacent to the outlined areas indicate MitoTracker-positive proportion of cells and are summarized in the graph on the right. Flow cytometry analyses of BODIPY^493/503^ (G) and 2-NBDG (H) in T_FH_ and T_H_1 cells from the spleens of vehicle-treated or tamoxifen-treated mice in experiments described in panel A. ns, not significant;*, *P* < 0.05 (unpaired two-tailed *t* test). Data are representative of two independent experiments with at least 5 mice per group (for B to H, error bars are SEMs).

Next, we sought to explore the role of EZH2 in the late differentiated phase/memory phase of the CD4 T cell response to acute viral infection. To this end, *Ezh2*^fl/fl^ERT2-Cre mice were infected with LCMV Armstrong, administered tamoxifen from day 9 to day 12, and analyzed at day 55 postinfection (Fig. 7A). We found that the numbers and proportions of CD4 cells and mTORC1 activity were unaffected in EZH2-deficient CD4 T cells (Fig. 7B to D). As a result, mitochondrial function, lipid synthesis, and glucose uptake were comparable between vehicle-treated groups (EZH2 WT) and tamoxifen-treated groups (EZH2 KO) (Fig. 7E to H). To sum up, these results revealed that the EZH2-mTOR axis orchestrates the proliferation of early-activated but not late-differentiated or memory CD4 T cells during acute viral infection.

### EZH2 is the prerequisite for the recall response of virus-specific memory CD4 T cells.

**FIG 8:**
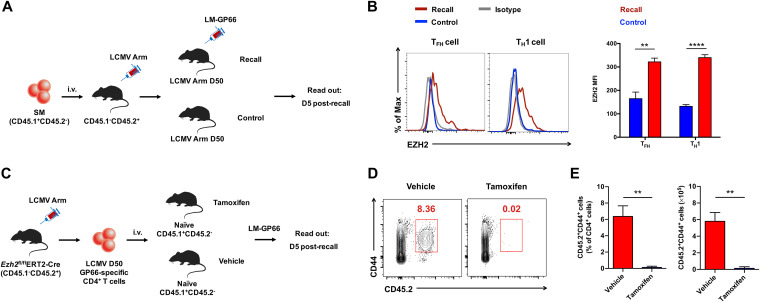
EZH2 is a prerequisite for the recall response of virus-specific memory CD4 T cells. (A) Experimental setup. (B) Flow cytometry analysis of EZH2 expression levels in SM T_FH_ and T_H_1 cells of recall group and control group in experiments described in panel A, which are summarized in the graph on the right. (C) Experimental setup. (D) Flow cytometry analysis of CD4 T cells in the spleens of recipients in experiments described in panel C. Numbers adjacent to the outlined areas indicate transferred LCMV GP66-specific CD4 T cells; cell number and frequency are summarized in E. **, *P* < 0.01; ***, *P* < 0.001 (paired two-tailed *t* test). Data are representative of two mice per group.

The generation of the CD4 T cell recall response is important to protect the host from reinfection. To explore whether the CD4 T cell recall response also requires EZH2, we first transferred naive SM cells into WT C57BL/6J recipient mice and subsequently infected the recipients with LCMV Armstrong strain virus. On day 50 postinfection, when CD4 T cell memory has been established, recipients were rechallenged with Listeria monocytogenes expressing the LCMV glycoprotein epitope I-A^b^GP66-77 (LM-GP66). Five days after LM-GP66 infection, EZH2 expression by memory SM CD4 T cells was measured (Fig. 8A). As indicated in Fig. 8B, we found enhanced EZH2 expression in recalled SM CD4 T_FH_ cells and T_H_1 cells compared to basal EZH2 expression levels in unrecalled ones, suggesting EZH2 might play an important role in regulating CD4 T cell recall responses in acute viral infection. To prove this hypothesis, we infected *Ezh2*^fl/fl^ERT2-Cre mice with LCMV Armstrong virus. On day 50 postinfection, LCMV virus-specific memory CD4 T cells were sorted from the infected *Ezh2*^fl/fl^ERT2-Cre mice and transferred into congenic naive C57BL/6J recipients. Then, the recipient mice were treated with tamoxifen (or vehicle) to acutely delete EZH2 in the transferred memory CD4 T cells followed by an LM-GP66 rechallenge (Fig. 8C). On day 5 after LM-GP66 infection, the transferred virus-specific CD4 T cells with tamoxifen-induced EZH2 deficiency were barely detectable, while vehicle-treated mice showed a robust memory CD4 T cell response upon infection (Fig. 8D and E). Taken together, these results indicated a crucial role of EZH2 in the CD4 T cell recall response.

## DISCUSSION

In response to acute viral infection, virus-specific CD4 T cells differentiate into either T_FH_ or T_H_1 cells (16–18). Of these, T_FH_ cells provide essential assistance to cognate B cells and thus initiate the germinal center reaction, which drives the rapid production of high-affinity virus-specific antibodies and long-term humoral immune memory (24); T_H_1 cells produce crucial cytokines such as gamma interferon (IFN-γ), resulting in macrophage activation and enhanced killing of intracellular virus (25). Upon activation, CD4 T cells with abundant EZH2 expression adopt the T_FH_ cell fate; while CD4 T cells with modest EZH2 become T_H_1 cells (8). As an epigenetic regulator, EZH2 specifically stabilizes the chromatin accessibility of a cluster of T_FH_-lineage associated genes, especially *Bcl6* (8). Additionally, EZH2 is recruited to directly activate the transcription of *Bcl6* (9), thus priming the early differentiation of T_FH_ cells. In addition to the crucial role of EZH2 in T_FH_/T_H_1 cell differentiation, the present study also found that EZH2 is a prerequisite for the efficient expansion of CD4 T cells (including both T_FH_ and T_H_1 cells) during acute viral infection. In the absence of EZH2, both virus-specific CD4 T cell types show defective cell expansion, which is accompanied by impaired mTOR signaling activity and, consequently, compromised cellular metabolism. Thus, the present research further extends the role of EZH2 in antiviral CD4 T cell responses.

The regulatory function of EZH2 on mTOR signaling has been reported in many cell types. For instance, in neurons, EZH2 promotes mTOR signaling by suppressing the mTOR inhibitor Pten during fear memory reconsolidation (26). In tumor cells, EZH2 epigenetically represses negative regulators of mTOR (e.g., TSC2 and RHOA), thus inhibiting tumor cell autophagy and accelerating tumorigenesis (27). Considering mTOR as a central hub that senses and integrates environmental cues (11), it is not implausible to speculate that EZH2 functions as a regulator in cellular metabolism by fine-tuning mTOR signaling activity. Indeed, numerous studies reveal a pivotal role of EZH2 in regulating metabolic activities such as glycolysis, lipid synthesis, and mitochondrial function (28–31). Here, we also found that EZH2 is indispensable for mTOR signal activation in CD4 T cells during acute viral infection. Ablation of EZH2 leads to poor cellular metabolism and altered gene signatures related to oxidative phosphorylation, the electron transport chain, ATP synthesis, glycolysis, the TCA cycle, and fatty acid synthesis. With EZH2 deficiency-induced inadequate energy support, the CD4 T cell response to acute viral infection is largely diminished.

Naive CD4 T cells are activated with immunological inputs in the forms of TCR engagement, costimulatory receptor ligation, and cytokine stimulation, which instruct the differentiation of a distinct T_H_ subset with context-specific effector functions (32). Consistent with previous studies (8, 23), we found that naive CD4 T cells upregulate EZH2 expression upon TCR stimulation in both *in vivo* and *in vitro* assays. Particularly, during the CD4 T cell response to acute viral infection, EZH2 spikes within 3 days after infection and declines to a basal level comparable to that in the naive state at day 8 postinfection. An analysis of mTOR activity in virus-specific CD4 T cells revealed a similar dynamic pattern: mTOR signaling reaches a peak at day 2 postinfection and drops to a baseline level at day 8 after infection (33). In this scenario, TCR stimulation-trigged EZH2 upregulation seems to cooperate with mTOR signaling in the regulation of metabolism and cell expansion of early-activated CD4 T cells. The early-phase coupling of EZH2 and mTOR signaling is further evidenced by a limited effect of EZH2 in regulating mTOR activity in late-differentiated or memory CD4 T cells, of which, the strength of TCR stimulation is largely declined due to the substantial clearance of viral antigens during an acute viral infection. Furthermore, recalled memory virus-specific CD4 T cells upregulate EZH2 protein expression, and those devoid of EZH2 failed to respond to the TCR reengagement, further highlighting the role of TCR-EZH2-mTOR in guiding the CD4 T cell response. In addition to TCR engagement, interleukin 2 (IL-2) stimulation is reported as an important upstream signal that activates mTOR and regulates the CD4 T cell response (13). Indeed, we observed that both LCMV-specific T_FH_ and T_H_1 cells downregulated the IL-2 receptor CD25 (data not shown), suggesting that reduced sensitivity to IL-2 signaling in EZH2-deficient CD4 T cells might be responsible for their blunted mTOR signaling.

As a histone methyltransferase, EZH2 participates in fostering T_FH_ and T_H_1 cell development by stabilizing the T_FH_ cell-related gene chromatin accessibility (8) and H3K27me3-dependent gene repression of *Gata3* and *Il10* (6), respectively. Meanwhile, H3K27me3-independent functions of EZH2 have also been reported. For example, EZH2 directly activates the transcription of *Bcl6* and promotes T_FH_ cell differentiation (9), phosphorylation of EZH2 activates STAT3 (34), which regulates the ability of Bcl6 to repress target genes and thus preserves T_FH_ cell differentiation (35), and EZH2 binds to the *Tbx21* promoter and activates *Tbx21* transcription, thus fostering T_H_1 cell differentiation (36). Hence, the mechanisms underlying the abolished EZH2-deficient CD4 T cell expansion in this study may also be supplemented by EZH2-mediated H3K27me3-dependent and -independent regulations of hub transcriptional factors (e.g., Bcl6, T-bet, and STAT3) that dictate CD4 T cell differentiation and proliferation. In addition to transcriptional factors, EZH2-mediated H3K27me3 modifications of cell cycle inhibitors also preserve T cell proliferation (37). In this regard, we also observed enhanced gene expressions of cell cycle inhibitors (e.g., *Cdkn2a*) in the scenario of EZH2 deficiency (data not shown), which further supports the role of EZH2 in regulating the CD4 T cell response.

In conclusion, our present study demonstrates that the epigenetic regulator EZH2 is integral for CD4 T cell expansion during acute viral infection. Mechanistically, EZH2 is involved in initiating mTOR signaling and, consequently, regulating gene signatures closely related to cell metabolism. Additionally, metabolic regulation of EZH2 is strictly limited in early-activated CD4 T cells rather than late-differentiated or memory CD4 T cells. These findings provide potential avenues for strategies targeting EZH2 to improve the efficacy of a CD4 T cell-based virus vaccine and to cure diseases associated with aberrant CD4 T cell responses.

## MATERIALS AND METHODS

### Mice, virus, and tamoxifen treatment.

SMARTA mice were provided by R. Ahmed (Emory University). The *Ezh2^fl/fl^Cd4*-Cre transgenic, ERT2-Cre transgenic, and C57BL/6J (CD45.1 and CD45.2) mice were from Jackson Laboratories. The Lymphocytic choriomeningitis virus (LCMV) Armstrong strain was a gift from R. Ahmed (Emory University). For the establishment of the acute viral infection mouse model, 2 × 10^5^ PFU of this strain were injected intraperitoneally into 60 to 10-week-old mice of both sexes without “blinding.” Bone marrow chimera mice were infected after 2 months of reconstitution. Tamoxifen (T5648; Sigma-Aldrich) was injected intraperitoneally into mice (1 mg/mouse/day for 4 days). Infected mice were housed in accordance with institutional biosafety regulations of the Third Military Medical University. Mouse experiments were performed under the guidelines of the Institutional Animal Care and Use Committees of the Third Military Medical University.

### Flow cytometry and antibodies.

Flow cytometry data were acquired with a FACSCanto II (BD Biosciences) and analyzed by using FlowJo software (Tree Star). The major histocompatibility complex (MHC) class II (I-A^b^) tetramer of LCMV epitope of GP66-77 was provided by Rafi Ahmed (Emory University). MHC II GP66-77 tetramer staining was performed by incubating cells with the tetramer for 1 h at 37°C. Staining of surface markers CD4 (RM4-5; BioLegend), CD44 (IM7, BioLegend), CD45.1 (A20; BioLegend), and CD45.2 (104; Biolegend) was performed in phosphate-buffered saline (PBS) containing 2% fetal bovine serum (wt/vol) on ice. The tertiary CXCR5 staining and the phosphorylated mTOR signaling protein (pS6 and p4E-BP1) staining have been described previously (14, 38). To assess metabolism, splenocytes were first stained with surface marker antibodies and then stained with MitoTracker (M7514; Thermo Fisher Scientific), CellROX (C10422; Thermo Fisher Scientific), BODIPY^493/503^ (D-3922; Invitrogen), and 2-NBDG (N13195, Invitrogen) in PBS at 37°C for 30 min. The *in vitro* cell proliferation assay was performed with a CellTrace CFSE cell proliferation kit (C34554, Invitrogen) according to the manufacturer’s instructions.

### Immunofluorescence.

Immunofluorescence staining was performed as described previously (18). Briefly, sorted cells were transferred to 12-mm coverslips (354085; BD Biosciences) in a 12-well plate (2 × 10^4^ cells per well). Fixed and permeabilized, the cells on coverslips were then stained for EZH2 (5246 and 9733; Cell Signaling Technology). The nucleus was defined by using DAPI (4,6-diamidino-2-phenylindole) (D9542; Sigma-Aldrich).

### Adoptive transfer.

For adoptive transfer, 5 × 10^5^ (for analysis on day 2.5) or 1 × 10^4^ (for analysis on day 8 or day 55) CD45.1^+^ naive SMARTA cells were adoptively transferred into CD45.2^+^ recipients. On the following day, the recipients were infected intravenously with 1 × 10^6^ PFU LCMV Armstrong (day 2.5) or intraperitoneally with 2 × 10^5^ PFU LCMV Armstrong (day 8 or later).

### Sources for reference microarray data.

Reference microarray data of EZH2 WT and EZH2 KO CD4 T cells from LCMV Arm-infected mice (day 8) were obtained from Gene Expression Omnibus (GEO) (GSE110458) (8).

### Bone marrow chimera.

Bone marrow cells harvested from *Ezh2*^fl/fl^ERT2-Cre (CD45.2^+^) mice and C57BL/6J wild-type (CD45.1^+^) mice were mixed at a ratio of 4:6 (2 × 10^6^ in total) and then injected intravenously into lethally irradiated (2 doses of 550 rads each) C57BL/6J wild-type recipients (CD45.1^+^). Two months later, the bone marrow recipient mice were infected with LCMV Armstrong.

### Mitochondrial oxygen consumption.

On day 8 post LCMV Armstrong infection, 2 × 10^5^ virus-specific CD4 T cells were sorted from the spleens of *Ezh2*^fl/fl^ or *Ezh2*^fl/fl^*Cd4*-Cre mice and seeded in each well of an XFp cell culture miniplate in 180 μl XF assay medium. Subsequently, the XFp miniplate was placed in a 37°C non-CO_2_ incubator for 30 min to equilibrate the temperature. Oxygen consumption rate (OCR) was measured using a Seahorse XFp Extracellular Flux analyzer with a XFp Cell Mito Stress test kit (Seahorse, Bioscience). OCR was determined at four levels: basal respiration with no additions and after adding oligomycin (1 μM), carbonyl cyanide 4-(trifluoromethoxy) phenylhydrazone (FCCP; 1.5 μM), and rotenone/antimycin A (0.5 μM). Then, ATP production and spare respiratory capacity were analyzed and compared between EZH2 WT and EZH2 KO virus-specific CD4 T cells.

### Statistical analysis.

Statistical analysis was conducted with Prism 6.0 (GraphPad). An unpaired two-tailed *t* test with 95% confidence interval was used to calculate *P* values, and a one-way analysis of variance (ANOVA) (Tukey’s multiple comparisons) was used for the longitudinal analysis. For bone marrow chimera experiments, a paired two-tailed *t* test with 95% confidence interval was used for the calculation of *P* values.
